# Penile strangulation in a patient with Parkinson's disease: a case report

**DOI:** 10.1186/1757-1626-2-9379

**Published:** 2009-12-22

**Authors:** Germar M Pinggera, Renate Pichler, Peter Rehder, Andrea Kerschbaumer, Alexander Buttazzoni, Florian Zangerl, Michael Mitterberger

**Affiliations:** 1 Department of Urology, Medical University Innsbruck, Anichstrasse 35, Innsbruck, 6020, Austria

## Abstract

**Introduction:**

Aberrant sexual behaviour such as hypersexuality or exhibitionism has been reported in patients with Parkinson's disease and its therapy.

**Case presentation:**

We report a case of a 67-year-old man with a 10-year history of Parkinson's disease, currently under therapy with L-dopa and bromocriptine, who presented with his wedding ring constricting the base of his penis. The ring could be removed with a ring cutter without complications.

**Conclusion:**

The present literature concerning sexual dysfunction in Parkinson's disease and dopaminergic therapy is discussed. Doctors who treat these patients should be aware of the problem of hypersexuality associated with dopaminergic therapy.

## Introduction

Penile strangulation with constricting devices is not uncommon[1]. Sexual dysfunction in patients with Parkinson's disease and its therapy has been reported[2]. We report the first case of a Parkinson's patient, who presented with his wedding ring constricting the base of the penis because of autoerotic stimulation.

## Case presentation

**Figure 1:**
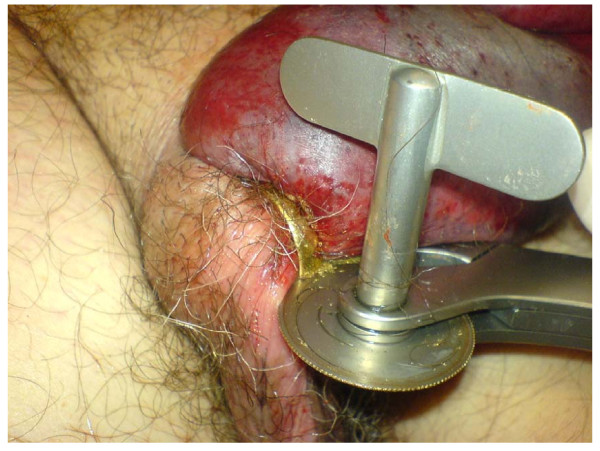
**Removal of the wedding ring by ring cutter**. Notice the distal penile oedema.

A 67 year old caucasian man presented to the Urological Department with his wedding ring strangulating the base of his penis for 11 hours. The patient had a 10-year history of Parkinson's disease and was currently under therapy with L-dopa and bromocriptine. Under these medications the patient lived inconspicuously with his family; no particular psychosocial history, especially no sexual impulsive disorders or deviations have been reported from his wife. The patient realised and expressed his shame of the situation but on the other hand was unable to give any clear information about the cause of his autoerotic behaviour. His wife could not exclude that an overdose of medication was accidentally administered. At presentation his penis was grossly swollen and initial attempts to remove the ring failed. The patient was taken to the operating room and the ring was removed with a ring cutter without complications under local anaesthesia (Fig 1). Swab soaked with cold saline were used to avoid burns from sparks or excessive heating. Intravenous antibiotic therapy was started and 24 hours later the oedema had subsided and the patient was discharged. No further sexual impulsive disorders were reported after this occurrence after confirming correct dosage of medication.

## Discussion

Studies showed that sexual function is commonly reduced in patients with Parkinson's disease (PD)[2,3]. Also, it is known that Levo-dopa or other dopaminergics in the therapy of PD may cause hypersexuality[4,5]. Hypersexuality appears simultaneously with hyperkinesias under treatment with L-dopa and bromocriptine or in a dose-dependend way with bromocriptine and pergolide. Further studies showed that this hypersexuality is caused by an increase in libido, rather than frontal lobe disinhibition [2-5].

In another study the influence of PD on sexuality and partnership was investigated in 2099 patients[6]. Sexual dysfunction occurred in affected women and men with a reduction of sexuality. The patients mentioned specific symptoms of PD and medication as factors influencing their sexuality. Dopaminergic drugs had an influence on their sexual function in 30% of the women and in 64% of the men. Hypersexuality under L-dopa therapy was reported in 10-20% of male and in 40% of female patients.

Three causes are postulated to encounter disturbing hypersexuality. First the use of alternative dopamine agonists, which normally should have no enhancing effect on sexuality. Second the initiation of an anti-psychotic therapy with atypical neuroleptics like clozapine. And third the use of antiandrogens[2,4-6].

**Figure 2:**
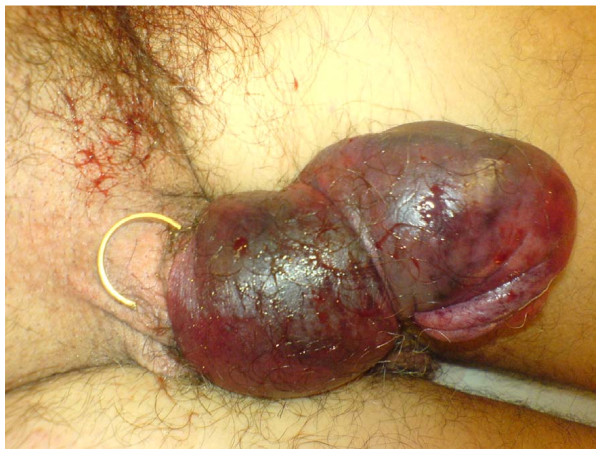
**Appearance of the penis after removal of the ring**.

In contrast to the PD and its therapy related sexual dysfunction the penile strangulation and its therapy is straight forward. Various devices have been described to cause strangulation of the male external genitalia[1]. The constriction impedes the venous and lymphatic return causing distal oedema eventually leading to ischemia (Fig 2). Also different methods have been described to remove the strangulation device and most patients recover without any major problems[7].

In conclusion dopaminergic therapy of PD has the potential risk of sexual hyperfunction. The attending physician should be aware of increased sexuality or reduced control of behaviour in PD therapy and to manage this by counteractive therapeutic strategies. Critical situations might be caused by change of medication or dosages, keeping in mind that patients might alter intake of medication independently.

## Competing interests

The authors declare that they have no competing interests.

## Authors' contributions

All authors contributed to patient care and draft.

## Consent

Written informed consent was obtained from the patient for publication of this case report and accompanying images. A copy of the written consent is available for review by the journal's Editor-in-Chief.
